# The Evolution of Southern Ocean Net Primary Production in a Changing Climate: Challenges and Opportunities

**DOI:** 10.1111/gcb.70653

**Published:** 2025-12-15

**Authors:** Alessandro Tagliabue, Thomas Ryan‐Keogh, Alex Baker, Thomas S. Bibby, Chris Follett, Maeve C. Lohan, Alberto Naveira‐Garabato, Daniel J. Mayor, Angela Milne, C. Mark Moore, Simon Ussher

**Affiliations:** ^1^ School of Environmental Sciences University of Liverpool Liverpool UK; ^2^ Southern Ocean Carbon‐Climate Observatory, CSIR Cape Town South Africa; ^3^ National Oceanography Centre Southampton UK; ^4^ School of Environmental Sciences University of East Anglia Norwich UK; ^5^ School of Ocean and Earth Science University of Southampton Southampton UK; ^6^ Biosciences, University of Exeter Exeter UK; ^7^ School of Geography, Earth and Environmental Sciences University of Plymouth Plymouth UK

**Keywords:** climate change, GEOTRACES, modelling, phytoplankton, primary production, Southern Ocean, zooplankton

## Abstract

Net primary production in the Southern Ocean plays a critical role in governing ecosystem production, the biological carbon pump, and global biogeochemical cycles. Recent work has advanced our understanding of novel factors regulating Southern Ocean net primary production and the regional physiological adaptations employed by Southern Ocean phytoplankton in terms of their photosynthetic strategies and resource acquisition. Here we assess trends in Southern Ocean net primary production from different remote sensing algorithms and bgc‐Argo floats to compare them to the latest Earth System Models used to forecast future changes under three different future climate scenarios. Overall, remote sensing and bgc‐Argo floats indicate net primary productivity in the Southern Ocean is declining at basin scale. This contrasts with the Earth System Models that display muted contemporary trends and consistent increases in net primary production that are relatively robust across SSP2‐45, SSP3‐70, and SS5‐85. This mismatch in trends suggests low confidence in these projected net primary production changes, with implications for assessments of changes in ecosystem services. Despite their coherence in terms of net primary production trends, Earth System Models show large disagreement in the relative role of different drivers, suggesting we lack sufficient mechanistic understanding. Improved knowledge of the role of manganese alongside iron and the coupled responses of phytoplankton and zooplankton through the integration of observations and experiments into a new generation of models is necessary to deliver confident forecasts of Southern Ocean ecosystem change. Advancing knowledge in these areas is an important priority for future research in the region and provides context for policy discussions around the marine protection of Antarctic ecosystems that depend on sufficiently confident projections of climate change impacts.

## 1 Introduction

Net primary production (NPP) is the gross fixation of inorganic CO_2_ into organic carbon during photosynthesis, minus autotrophic respiration, which supports marine ecosystems and catalyzes upper ocean biogeochemical cycles. By building up the standing stock of particulate organic carbon, NPP primes the ocean's biological pump and thus regulates the contribution of ocean biology to the global carbon cycle, as well as supplying organic matter to higher trophic levels and the ocean interior. These roles are particularly important in the Southern Ocean, which is the hub of the global ocean circulation, where NPP changes have important local and far‐field impacts, particularly on ocean biogeochemistry and the carbon cycle (Meredith et al. 2019). Replete in the ‘major nutrients’ nitrogen and phosphorus thanks to strong upwelling generating the highest global surface concentrations of their major bioavailable forms of nitrate and phosphate, respectively, the Southern Ocean is deficient in iron (Fe) (Browning and Moore 2023), which is required for a range of cellular processes, including photosynthesis, respiration, and acquisition of oxidized nitrogen forms (Raven 1990). In addition, the role of an additional micronutrient, manganese (Mn), has also been evidenced in recent years (Balaguer et al. 2022; Browning et al. 2021; Hawco et al. 2022; Latour, Strzepek, et al. 2023; Wyatt et al. 2023) due to its low availability and essential role in the oxygen‐evolving complex of photosystem II (PSII), a key component of the photosynthetic apparatus (Raven 1990).

The biogeochemical context of the Southern Ocean (high major nutrients and low Fe and Mn) is unique in the global ocean and occurs in parallel with a range of adaptations displayed by the region's phytoplankton community. While Fe is well understood to be depleted in the Southern Ocean, Mn concentrations are significantly lower than in other ocean basins, with the usual surface maxima not observed (Latour et al. 2021; Middag et al. 2013). Notably, other Fe‐limited regions display much higher surface Mn concentrations. Field and experimental work have demonstrated that Southern Ocean phytoplankton display atypical attributes, relative to temperate organisms, in their nutrient acquisition and photo‐adaptive traits (Andrew et al. 2023; Strauss et al. 2023; Strzepek et al. 2012; Strzepek et al. 2011; Sunda and Marchetti 2024) that have likely arisen in response to the parallel depletion of Fe and Mn in the region (Hawco et al. 2022). Thought to economize their Fe demands, Southern Ocean phytoplankton are typified by large photosynthetic antennae, whereby large Fe‐free complexes of pigments are coupled to relatively few Fe‐requiring reaction centers (Strzepek et al. 2019; Strzepek et al. 2012). Unlike temperate phytoplankton, which adapt to gradients in Fe by increasing the relative abundance of the relatively Fe‐poor PSII to the relative Fe‐rich PSI (Strzepek and Harrison 2004), Southern Ocean phytoplankton do not adjust the balance between PSII and PSI by the same degree (Strzepek et al. 2019), possibly to avoid the generation of Mn stress (Hawco et al. 2022). Lastly, Southern Ocean phytoplankton display a much greater ability to obtain Fe bound to strongly complexing siderophores (Strzepek et al. 2011) and deploy Fe‐free proton‐pumping rhodopsins (Andrew et al. 2023; Strauss et al. 2023; Sunda and Marchetti 2024), which confers an additional competitive advantage in Fe‐poor waters.

Efforts to conserve Southern Ocean biodiversity in a changing climate and quantify the broader impacts of NPP changes on ocean biogeochemical cycles require the assessment of how NPP is affected by different future climate scenarios (Constable et al. 2023; Meredith et al. 2019). This is typically achieved using complex Earth System Models (ESMs) that account for the role of iron and other major nutrients on globally distributed ‘cosmopolitan’ phytoplankton functional types (e.g., ‘diatoms’, ‘nanophytoplankton’, ‘calcifying plankton’, ‘nitrogen fixing plankton’, etc.). At present, while global NPP changes from ESMs remain highly uncertain, the picture is more consistent in the Southern Ocean, with NPP predicted to rise under high emissions scenarios (Bopp et al. 2013; Leung et al. 2015; Tagliabue et al. 2021). This view of increasing NPP in a changing ocean is at odds with estimates of increasing iron stress over the past few decades that indicate ongoing declines in regional NPP (Ryan‐Keogh, Thomalla, Monteiro, and Tagliabue 2023). However, we lack a systematic assessment of Southern Ocean satellite‐based NPP trends and how they vary across the different mathematical algorithms used to derive NPP rates from remote sensing observations. Moreover, we do not have a good understanding of how ongoing trends from ESMs match those from remote sensing and how they depend, in turn, on different climate scenarios. This assessment is necessary to appraise the current confidence in future NPP trends in this critical region, especially urgent given the ongoing regime shifts in sea‐ice dynamics over recent years (Purich and Doddridge 2023) and extremes in regional warming.

In this manuscript, we present a comprehensive assessment of NPP changes in the Southern Ocean from six different remote sensing algorithms applied to the single European Space Agency Ocean Colour Climate Change Initiative (ESA OC‐CCI) (Sathyendranath et al. 2019) ocean colour dataset (Ryan‐Keogh, Thomalla, Chang, and Moalusi 2023). These trends are compared to those from BGC‐Argo floats and the historical and future trends from 14 CMIP6 ESMs that represent the coupled land‐atmosphere–ocean system for three different shared socio‐economic pathway (SSP) climate scenarios (SSP2‐45, SSP3‐70 and SSP5‐85: spanning low, medium and high emissions trajectories, respectively). We find that ongoing declines in regional NPP are most common in remote sensing‐based NPP algorithms and are consistent with shorter timescale trends derived from BGC‐Argo floats. However, these trends are inconsistent with those from ESMs that consistently project NPP increases. A deeper comparison of ESM trends in phytoplankton biomass and stresses in light and iron, for a subset of 5 ESMs, reveals a wide array of projected changes across the three scenarios that are not always closely coupled with NPP trends. We argue that the unique aspects of the Southern Ocean system need to be better understood from a new generation of inter‐disciplinary studies such that they can be integrated into a new generation of ESMs for climate projections.

## 2 Methods

For simplicity, we defined the Southern Ocean using a single latitudinal cut‐off of 40° S.

Remote sensing net primary production (NPP; mg C m^−2^ day^−1^) was calculated using the following six algorithms from three broad groups: the ‘vertically generalised production model’ (VGPM) suite: Behrenfeld‐VGPM (Behrenfeld and Falkowski 1997) and Eppley‐VGPM which uses the temperature coefficients from Eppley (Eppley 1972); the ‘carbon‐based production model’ (CbPM) suite: Behrenfeld‐CbPM (Behrenfeld et al. 2005) and Westberry‐CbPM (Westberry et al. 2008); and the ‘absoprtion‐based’ suite: ‘absorption‐based production model (AbPM)’ (Lee et al. 2011); and the ‘carbon, absorption and fluorescence euphotic’ resolving model (Silsbe‐CAFE) (Silsbe et al. 2016). The algorithms were applied to ocean colour remote sensing data from the ESA‐OC‐CCI data product (8‐day, version 6.0) (Sathyendranath et al. 2019), including chlorophyll‐a (Chl‐a; mg m^−3^), particulate backscattering (b_bp_; m^−1^), phytoplankton absorption (a_ph_; m^−1^), detrital absorption (a_dg_; m^−1^), attenuation coefficient (K_d_(λ490); m^−1^) and the spectral slope of backscattering (η; m nm^−1^) calculated following (Pitarch et al. 2019) (see Table S1 for complete details). Additional data products, see Table S1 for their sources, include photosynthetically active radiation (PAR; mol photons m^−2^ day^−1^), sea surface temperature (SST; °C), mixed layer depth (MLD; m—defined with a density criterion of 0.03 kg m^−3^), the nitracline depth (ZNO_3_; m—defined as the depth where nitrate + nitrite is 0.5 μM) and sea surface salinity (SSS). For a full description of how the remote sensing NPP data was generated and processed, please see (Ryan‐Keogh et al. 2025; Ryan‐Keogh, Thomalla, Chang, and Moalusi 2023).

Biogeochemical‐Argo (bgc‐Argo) data were downloaded from the Hermes GDAC (January 2025 Snapshot). Only quality‐controlled profiles, using flags 1, 2, 5, or 8 (Wong et al. 2024) from floats that were deployed with a PAR sensor, were included in the analysis. Briefly, the vertical grid was standardised from 1 to 1000 m, with a 1 m resolution in the upper 300 m and a 10 m resolution below that, where the data were interpolated onto this grid using a Hermite polynomial scheme with no extrapolation outside of the observed range (Schlosser et al. 2025). Quenching correction of Chl‐a fluorescence data was performed using the scheme from (Xing et al. 2018), which was converted to Chl‐a using a slope adjustment factor of 2 (Roesler et al. 2017). b_bp_ (λ700) data were additionally checked to remove negative values, noisy profiles, and profiles with anomalously high deep values (Dall'Olmo et al. 2022). The non‐algal proportion of the b_bp_ signal was removed (Stoer and Fennel 2024), converted to 470 nm with a spectral slope of −0.78, and finally to phytoplankton carbon (C_phyto_; mg C m^−3^) using the slope factor (12128) from (Graff et al. 2015). All data variables were cleaned and despiked using an 11‐point running median (Xing et al. 2018). Bgc‐Argo NPP was calculated using the VGPM and CbPM algorithms, where Westberry‐CbPM was modified as in previous work (Arteaga et al. 2022), at every depth interval (mg C m^−3^ day^−1^) before being integrated over the top 200 m to provide depth integrated NPP (mg C m^−2^ day^−1^).

Remote sensing trends of NPP were calculated by first excluding any pixel whose time series had less than 50% of the data available. The time series of each pixel was tested for normality using the D'Agostino‐Pearson test in the Scipy python package (Virtanen et al. 2020). Normally distributed time series trends were calculated using the Sci‐Kit Huber‐Regression (Pedregosa et al. 2011), with an epsilon value of 1.35 to detect outliers whilst ensuring maximum robustness (Huber 1981). If more than 50% of the time series was identified as an outlier, then no trend is reported. Non‐normally distributed time series trends were calculated using the non‐parametric Mann‐Kendall test (Hussain and Mahmud 2019). Trends were corrected for area weighting by determining the number of m^2^ per pixel as a function of latitude. Bgc‐Argo trends of NPP were performed using ordinary least‐squares regression of annual means for float profiles south of 40° S.

CMIP6 ESM data were obtained from the Earth System Grid Federation data server for the historical (1850–2014) and future (2015–2100) low emission (SSP2‐40), moderate emission (SSP3‐70), and high emission (SSP5‐85) scenarios. Data variables downloaded include depth integrated (to 100 m) NPP (‘intpp’; mol C m^−2^ day^−1^), phytoplankton biomass C_phyto_ (‘phyc’; mol C m^−3^), Fe limitation (LimFe), and light limitation (LimIrr) (see Table S2 for full details of models and available data). ESM C_phyto_ (mol C m^−3^) was integrated over the top 100 m, the same depth interval as NPP, to derive depth‐integrated C_phyto_ (mol C m^−2^). Fe limitation and light limitation (both represent the fractional limitation of the maximum growth rate, with a smaller number indicating greater limitation), which are available for each functional type in the surface only, were weighted according to the proportion of each functional type to the total phytoplankton biomass in the surface only. All variables were re‐gridded from their native model grid to a 1 × 1 degree resolution using climate data operators. Linear regression analyses were performed between the ΔNPP and ΔC_phyto_, ΔFe limitation and ΔLight limitation spatial patterns, where the Δ represents the difference between the end of the future period (2081–2100) and the end of the historical period (1995–2014).

## 3 Results and Discussion

### 3.1 Southern Ocean NPP Trends: Satellites, Floats and Models

**FIGURE 1:**
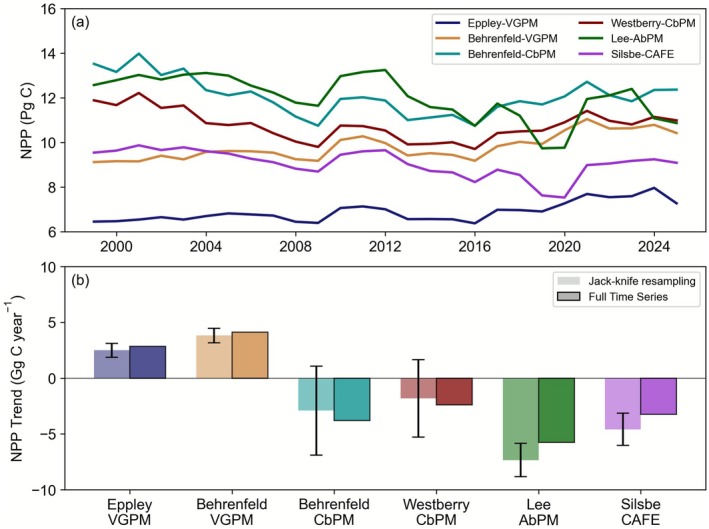
(a) Annual Southern Ocean NPP (Pg C) time series for six remote sensing algorithms: Eppley‐VGPM, Behrenfeld‐VGPM, Behrenfeld‐CbPM, Westberry‐CbPM, Lee‐AbPM, and Silsbe‐CAFE. (b) Long‐term NPP trends (Gg C year^−1^) averaged across the Southern Ocean for the full time series (darker shade) and a jackknife resampling of the full time series (1998–2024) as a moving 20‐year record with the mean trends ± the standard deviation reported (lighter shade).

**FIGURE 2:**
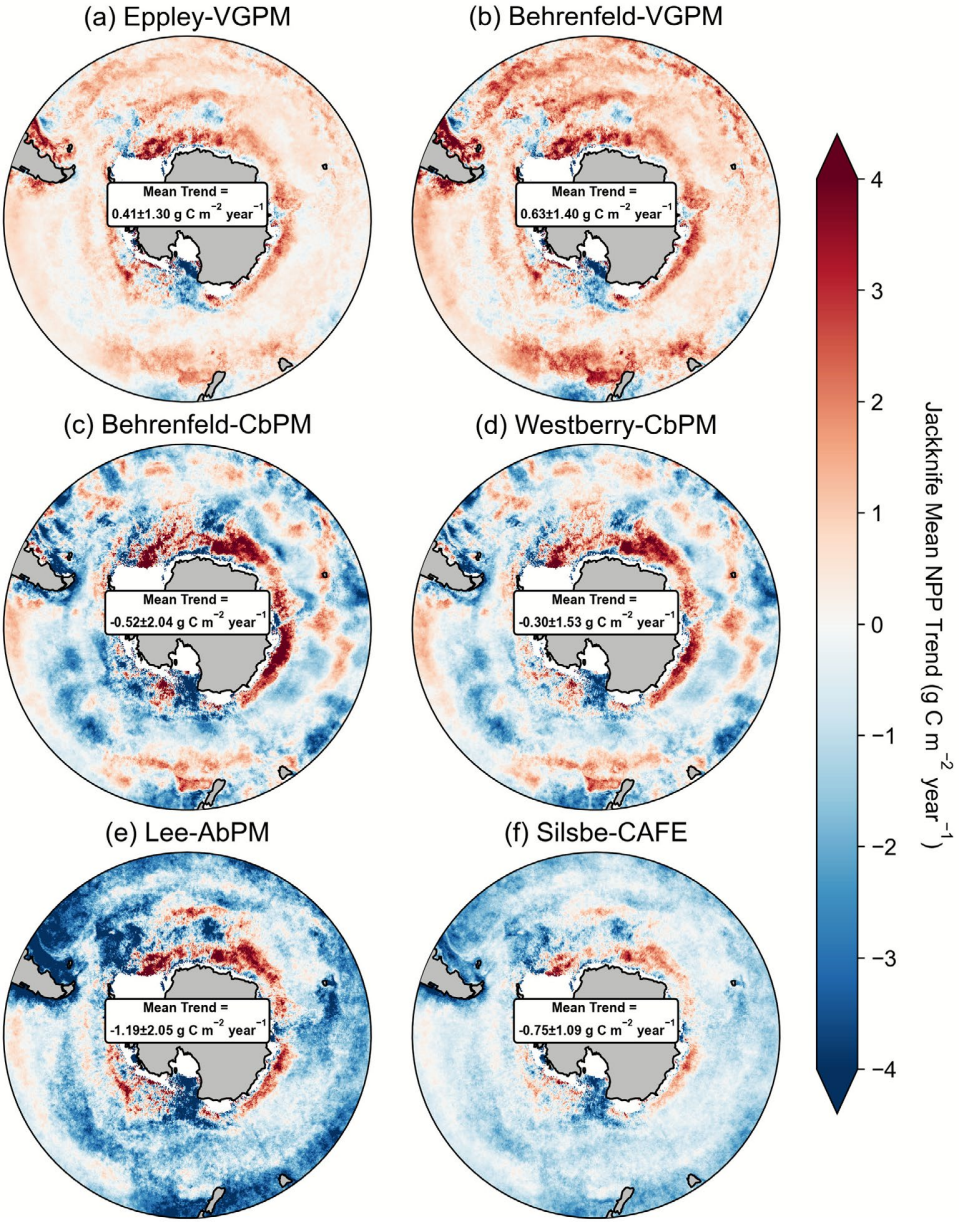
Long term mean Southern Ocean NPP trends (g C m^−2^ year^−1^) from a jack‐knife resampling of the time‐series (1998–2024) as a moving 20‐year record for the (a) Eppley‐VGPM, (b) Behrenfeld‐VGPM, (c) Behrenfeld‐CbPM, (d) Westberry‐CbPM, (e) Lee‐AbPM, and (f) Silsbe‐CAFE algorithms.

Notwithstanding a two‐fold span in the overall level of Southern Ocean NPP, there is a tendency to suggest regional NPP is decreasing for the majority of the remote sensing algorithms. Overall, Southern NPP varies between ~6 to ~13 Pg C across the six remote sensing algorithms for the 1998–2024 period (Figure 1a), with no systematic pattern associated with algorithm type or complexity. As shown in Figure 1b, the VGPM group of algorithms produces positive trends in Southern Ocean NPP between 1998 and 2024 of 2.5 ± 0.6 and 3.8 ± 0.6 Gg C year^−1^ (for Eppley‐VGPM and Behrenfeld‐VGPM, respectively), while the more complex carbon‐ and absorption‐based algorithms coalesce around declines of 2.9 ± 4.0 and 1.8 ± 3.5 (for CbPM‐Behrenfeld and CbPM‐Westberry, respectively) or 7.3 ± 1.5 and 4.6 ± 1.4 (for Lee‐AbPM and Silsbe‐CAFE, respectively) Gg C year^−1^. For the VGPM, CAFE, and AbPM algorithms, trends are relatively robust to varying the start/end period of the time series using a jackknife approach, while both CbPM algorithms are more sensitive to varying the start/end dates of the time series, with standard deviations 3–4 times greater (Figure 1b). Spatially, these regionally integrated trends are similar within the offshore Antarctic and sub‐Antarctic zones, but there are notable increases in NPP projected across all algorithms for the waters closest to Antarctica, especially off East Antarctica (Figure 2).

**FIGURE 3:**
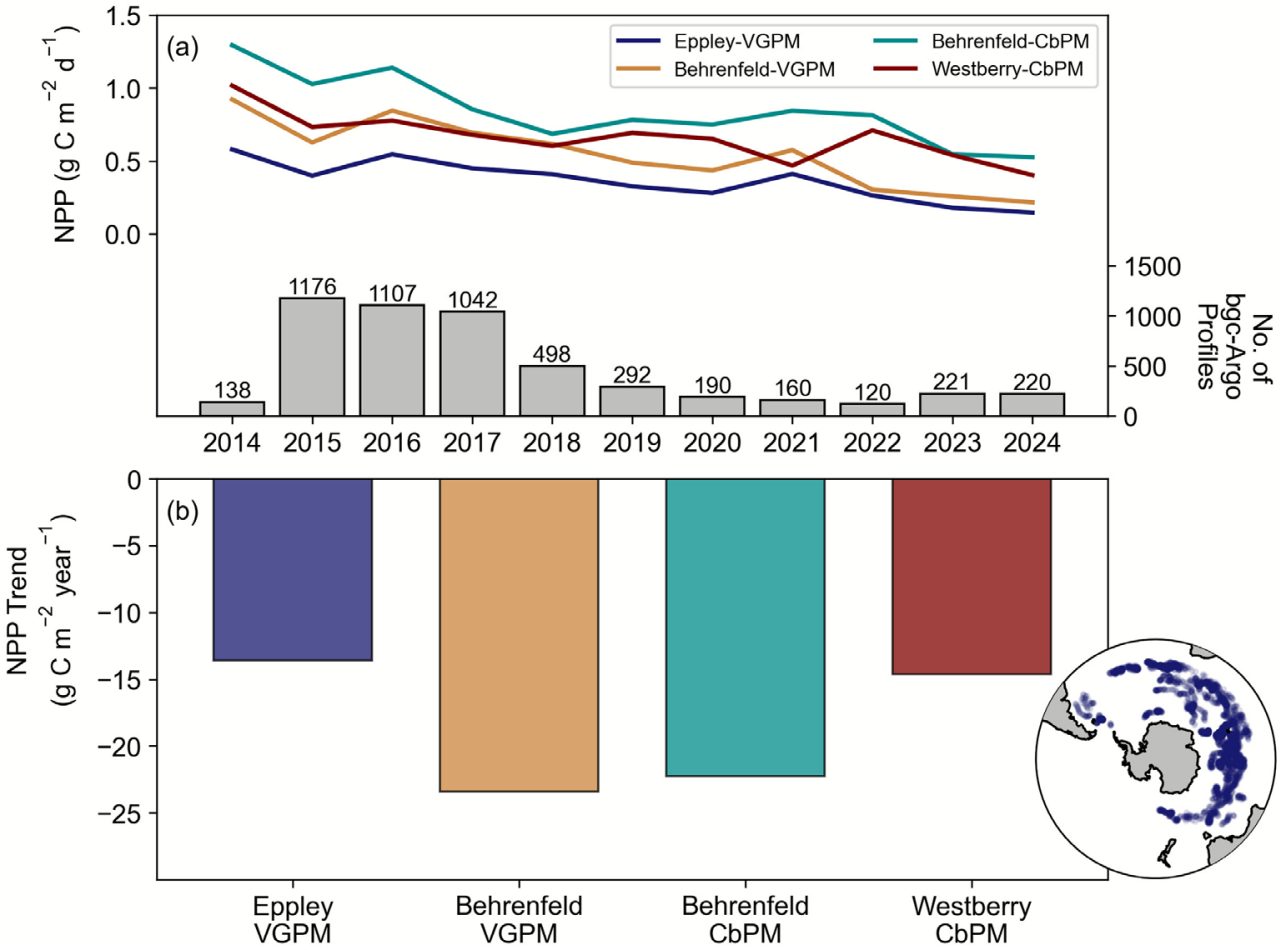
(a) Time series of NPP (g C m^−2^ day^−1^) from bgc‐Argo floats using the Eppley‐VGPM, Behrenfeld‐VGPM, Behrenfeld‐CbPM, and Westberry‐CbPM algorithms. The number of float profiles available per year is shown in the lower part of panel (a). (b) Overall trends in NPP (g C m^−2^ year^−1^) for the Eppley‐VGPM, Behrenfeld‐VGPM, Behrenfeld‐CbPM, and Westberry‐CbPM algorithms. Inset map shows the location of bgc‐Argo float profiles.

Albeit with shorter records, the NPP trends derived from Southern Ocean bgc‐Argo autonomous floats from 2014 to 2024 are also declining, consistent with the majority of the satellite algorithms. It is not possible to derive the most complex absorption‐based CAFE and AbPM algorithms using the available measurements from bgc‐Argo floats, but the VGPM and CbPM family can be quantified (Figure 3a). These calculations use the fully depth‐resolved PAR, fluorescence‐based chlorophyll, temperature, and C_phyto_‐based b_bp_ from bgc‐Argo. The ensuing bgc‐Argo NPP trends are broadly consistent across the four VGPM and CbPM algorithms and suggest declining regional NPP by between 14 and 23 g C m^−2^ year^−1^ (Figure 3b). The number of float profiles available to compute NPP has varied in time, from a high point of ~1000 profiles for a short period, with around 150–200 profiles more typical of the past 5 years (Figure 3a).

The mismatch in trends between remote sensing and bgc‐Argo for the VGPM algorithm is due to the opposite trend in chlorophyll. Remote sensing reflectance derived chlorophyll shows increases throughout the time series and over the shorter bgc‐Argo period (Figures S1 and S2), similar to that found in prior studies (Del Castillo et al. 2019). However, fluorescence‐derived chlorophyll trends from bgc‐Argo display a (much larger) decline over a shorter time period (Figures S1 and S2). This mismatch drives the difference in VGPM trends between the two platforms (the sign of other parameters is similar, Figure S2). Trends for the CbPM algorithm are similar between remote sensing and bgc‐Argo as there are fewer significant differences in input parameters (Figures S1 and S2). The spatial sampling of the system from bgc‐Argo is not even, but subsampling the remote sensing datasets with these locations can still provide a close approximation of the full trends (Table S3).

**FIGURE 4:**
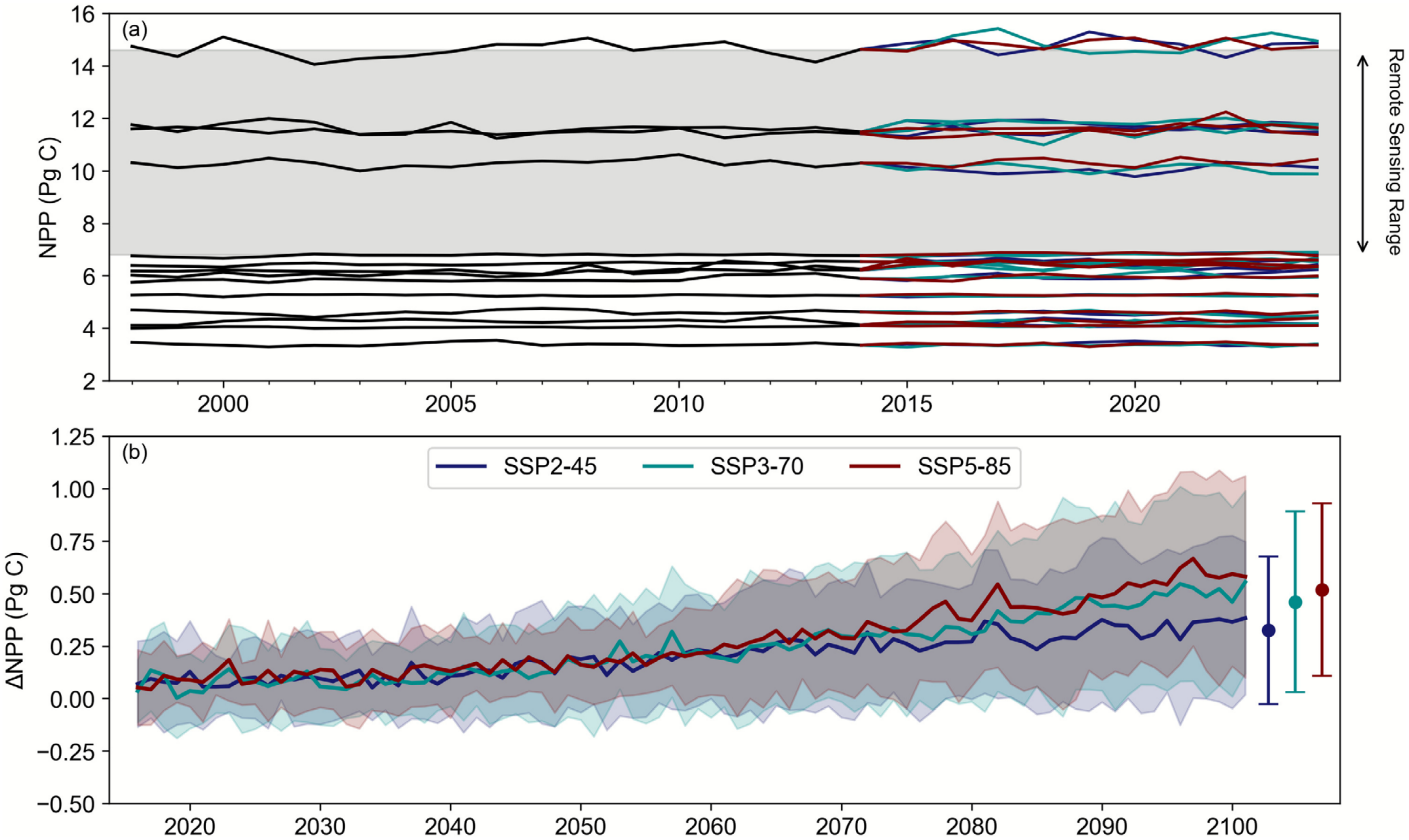
(a) Annual Southern Ocean NPP (Pg C) time series across 14 ESMs (b) Multi‐model mean long‐term NPP changes in NPP (Pg C) ± the standard deviation for the 14 ESMs for the SSP2‐45, SSP3‐70, and SSP5‐85 CMIP6 scenarios, relative to the end of the historical period (1995–2014). The overall multi‐model mean ± standard deviations for changes by 2081–2100 are shown adjacent.

**FIGURE 5:**
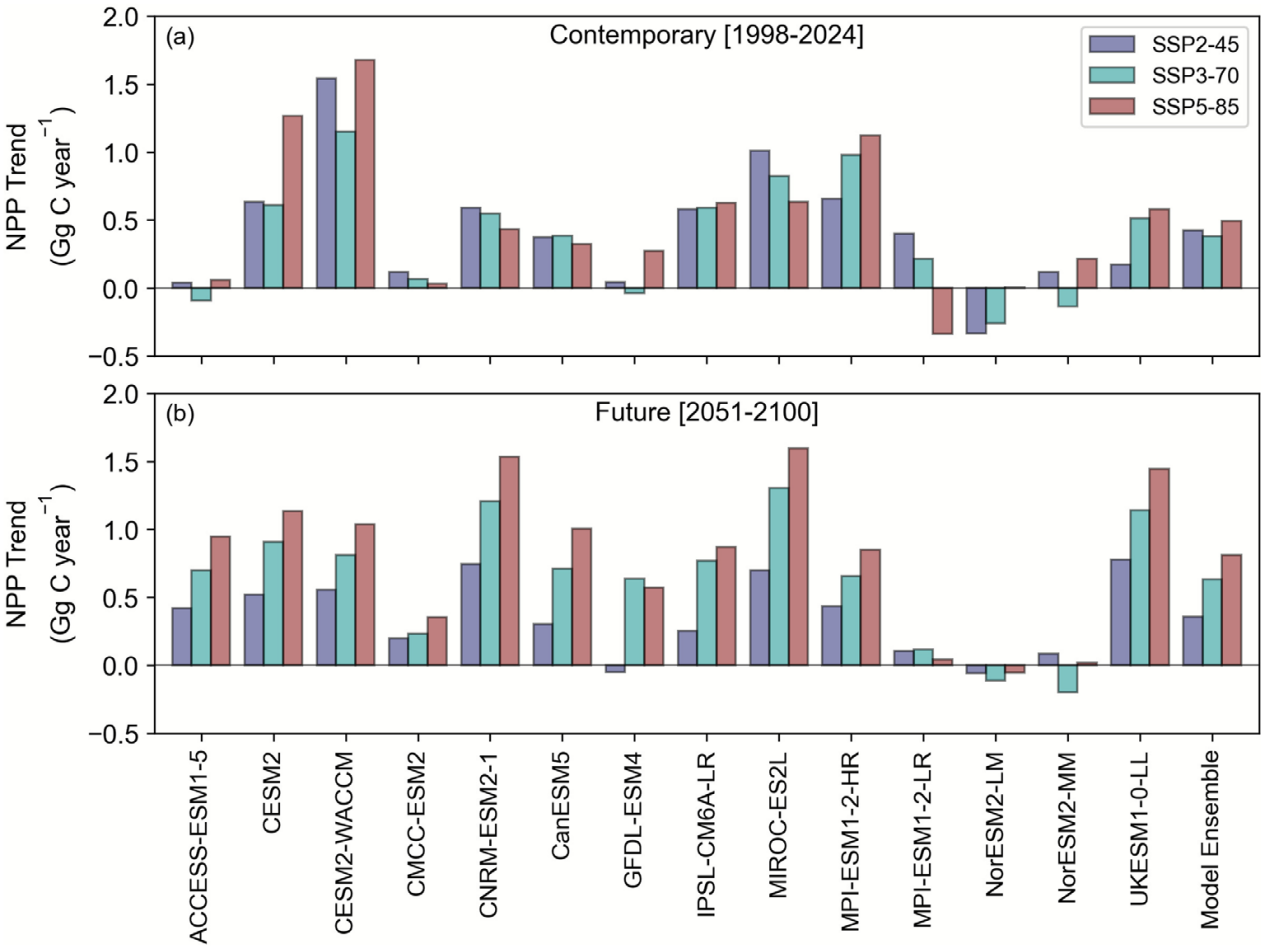
Annual Southern Ocean NPP trends (Gg C year^−1^) time series across 14 ESMs in the SSP2‐45, SSP3‐70, and SSP5‐85 CMIP6 scenarios for (a) the contemporary (1998–2024) and (b) future (2051–2100) periods. Trends were calculated following the same methods as remote sensing for Figure 2 (see Section 2).

While the absolute range in Southern Ocean NPP in CMIP6 models exceeds that from remote sensing, the future trends are relatively consistent and display a low scenario dependency. Over the historical period, there is a 5‐fold range in the mean state NPP in the Southern Ocean, from ~3 to almost 15 Pg C (Figure 4a), and only 5 models are able to fit within the range set by the remote sensing algorithms. Over the coming century, there is a projected trend of increasing NPP across the CMIP6 ensemble, with mean cumulative changes of 0.3 ± 0.4, 0.5 ± 0.4, and 0.5 ± 0.4 Pg C for the SSP2‐45, SSP3‐70, and SSP5‐85 scenarios, respectively, by 2081–2100 (Figure 4b). It is noteworthy that an overall increase in Southern Ocean NPP by 2081–2100 is a consistent feature across all scenarios, with the multi‐model mean ± standard deviations overlapping for the low, medium, and high emissions. Focussing on each specific model, we find that NPP trends over the historical and future periods are broadly similar but tend to be about 50% smaller than that seen for the remote‐sensing datasets (Figure 5). Notably, NPP trends show greater variability between models than between scenarios, highlighting that although there is overall model convergence on increasing NPP from the CMIP6 experiments, substantial across‐model uncertainty remains in the scenario‐specific response. This illustrates the overall poor consistency in the modelled NPP of this important region.

In summary, a consistent picture of declining NPP across the Southern Ocean emerges from most remote sensing algorithms and the shorter bgc‐Argo time series (which displays much larger trends). An exception is the Eppley‐VGPM and Behrenfeld‐VGPM algorithms, which show an increase using remote sensing data. However, both the Eppley‐VGPM and Behrenfeld‐VGPM algorithms produce a declining NPP trend from bgc‐Argo data (consistent with both types of CbPM algorithm). This mismatch is due to the different trends in derived chlorophyll between the platforms, which play a major role in driving the NPP calculated from the VPGM family of algorithms (Figure S1). CMIP6 models are also consistent, but display an opposite trend to the remote sensing and BGC‐Argo platforms, with NPP increasing and relatively low scenario sensitivity. Overall, NPP trends in CMIP6 models are an order of magnitude smaller than those from remote sensing, suggesting a major mismatch in both the sign and magnitude of regional change.

### 3.2 Trends in Key Controlling Factors of NPP

In the current generation of CMIP6 models, total primary producer biomass, Fe, and light are the major factors behind the projected NPP trends. Unfortunately, not all CMIP6 models archived these limitation terms at the monthly frequency necessary for this strongly seasonal region. Other potentially important components, such as zooplankton grazing of primary producer biomass (Laufkötter et al. 2015), are not archived at all. Nevertheless, we can explore the role of phytoplankton biomass, Fe limitation, and light limitation during the Austral summer for a subset of five ESMs across all three scenarios. Importantly, the NPP trends from this smaller subset are similar to those from the full CMIP6 ensembles, with projected cumulative NPP changes of 0.5 ± 0.2, 0.8 ± 0.2, and 0.8 ± 0.2 Pg C for SSP2‐45, SSP3‐70, and SSP5‐85, respectively, by 2081–2100.

**FIGURE 6:**
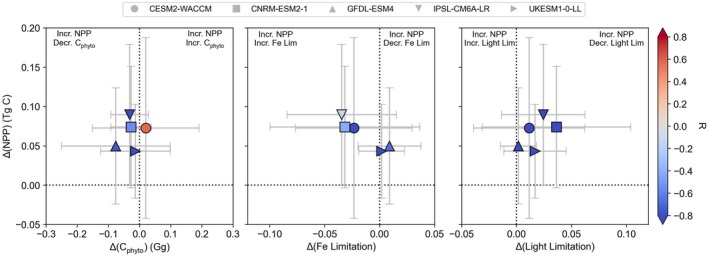
Synthesised assessment of how decadal scale changes in Southern Ocean NPP relate to those in phytoplankton biomass (C_phyto_), modelled ‘Fe limitation’ and modelled ‘light limitation’ for five CMIP6 ESMs between 2005–2014 and 2081–2100 for the SSP3‐70 scenario. Symbols represent the mean changes for each model, bars represent one standard deviation across the Southern Ocean region south of 40° S and the colour indicates the strength of the correlation coefficient from a linear regression in space and time between changes in each driver to the change in NPP for each model. Declining limitation terms indicate greater limitation of growth rates. Limitation axes are swapped to associate increasing NPP with decreasing limitation.

A regional analysis of NPP trends and their drivers reveals that while NPP trends are relatively coherent across ESMs, the underlying drivers are not. To disentangle the links between the trends in phytoplankton biomass, Fe limitation, and light limitation, we first addressed the regional patterns, focusing on SSP3‐70 (Figure 6, other scenarios can be found in Figures S3 and S4). The NPP data in Figure 6 summarizes the existing information (including prior assessments (Bopp et al. 2013; Leung et al. 2015)), whereby NPP generally increases across almost all of the region south of 40° S for the five models. This increase in NPP is, however, not always linked to an increase in phytoplankton biomass. The CESM2‐WACCM model displays a coherent positive link between increasing NPP and increasing phytoplankton biomass nearer the Antarctic continent (consistent across most models) and in the sub‐Antarctic (less agreement across models). All other models show more variability but tend to display a negative relationship between changes in NPP and phytoplankton biomass at the regional scale (Figure 6). This indicates that a simple explanation whereby increases in primary producer biomass drive NPP trends is not present in the majority of models.

Delving deeper into the factors explaining the NPP trends results in a complex response for Fe and a clearer signal of reduced light limitation. The modelled ‘Fe limitation’ displays a surprising link to projected NPP increases, with greater model ‘Fe limitation’ over time being the most common regional response across the five models. The modelled ‘Fe limitation’ diagnostic in these models is quantified as the fraction of maximum phytoplankton growth rates due to Fe, with a lower modelled ‘Fe limitation’ term indicating greater Fe limitation. Hence, the overall negative correlation between modelled ‘Fe limitation’ and NPP indicates that phytoplankton are achieving a lower fraction of their maximum growth rates, whilst also displaying greater NPP rates, which may appear counterintuitive. However, the fact that the diagnosed modelled ‘Fe limitation’ is based on the proportion of maximum growth rate achieved explains the observed response. As ocean warming enhances phytoplankton growth, the actual realised growth rate permitted by Fe may increase in absolute terms, even if Fe ends up supporting a smaller fraction of the now elevated maximum. Hence, the Fe‐limited growth rate may increase in absolute terms even if the model diagnostics also indicate greater modelled ‘Fe limitation’. This highlights a shortcoming of quantifying Fe control of phytoplankton growth through the model ‘Fe limitation’ diagnostics usually saved in models when there are large changes in maximum growth rates (e.g., due to warming). A better diagnostic to assess models would be via their Fe uptake, as shown in prior modelling work (Anugerahanti and Tagliabue 2024) or the Fe‐mediated growth rate, which links more closely to the impact of Fe on biological processes. However, these kinds of model diagnostic outputs are unfortunately not archived as part of CMIP6. Light limitation trends are clearer, with reduced light limitation associated with the greater NPP. That all being said, it is worth noting that the fractional projected changes in summertime limitation by Fe and light for SSP3‐70 are small (at most ±0.1) at the regional scale (Figure S5), in line with the small annual NPP trends shown in Figure 4.

## 4 Critical Knowledge Gaps

### 4.1 Integrating Fe and Mn Cycling Through Key Water Masses

One of the major advances in our understanding of the controls of Southern Ocean productivity in recent years has been the emergence of Mn as an important limiting nutrient alongside Fe. A key prerequisite to understanding the Fe and Mn cycles, and thereby their impacts on the magnitude and patterns of NPP, is to link external sources and internal cycling to the prevailing physical processes and pathways in the Southern Ocean. Amongst these, two sets stand out as most likely to be important in advancing our knowledge of the coupled Fe‐Mn biogeochemical system. First, the export of upper‐ocean waters from continental margins into the wider Southern Ocean will shape the distributions of both elements in offshore waters. Not only are these waters characteristically affected by sediment supply of Fe and Mn (Annett et al. 2012; Latour, van der Merwe, et al. 2023) (see Section 4.2), but their properties often reflect the integrated effects of sea ice formation and melt, and glacial meltwater from Antarctic glaciers and icebergs (Biddle et al. 2019). Notably, the export of Antarctic shelf waters into the open Southern Ocean is a highly heterogeneous process, focused on hotspots at which the continental shelf break topography or ambient wind forcing are conducive to intense offshore upper‐ocean transport (Brearley et al. 2019; Dawson et al. 2023). Second, a large fraction of upper‐ocean waters exported from the Antarctic continental shelves is injected into Winter Water (WW), the water mass occupying the winter mixed layer across the Southern Ocean to the south of the Polar Front (Evans et al. 2018; Spira et al. 2024). Further to modification by Antarctic shelf waters, WW is cooled and freshened by additional offshore melting of sea ice and icebergs, as well as being additionally transformed by the entrainment of upwelling Circumpolar Deep Water into the seasonally deepening winter mixed layer (Evans et al. 2018). As such, WW is likely to be a pivotal water mass in the Fe and Mn cycles, in that it integrates inputs from several candidate shelf sources, transports those inputs equatorward across the Antarctic Circumpolar Current, and potentially makes them available to primary producers as WW is re‐entrained into the mixed layer in subsequent winters (Evans et al. 2018; Spira et al. 2024).

### 4.2 Reducing Uncertainty the Water Column Cycling of Both Fe and Mn

**FIGURE 7:**
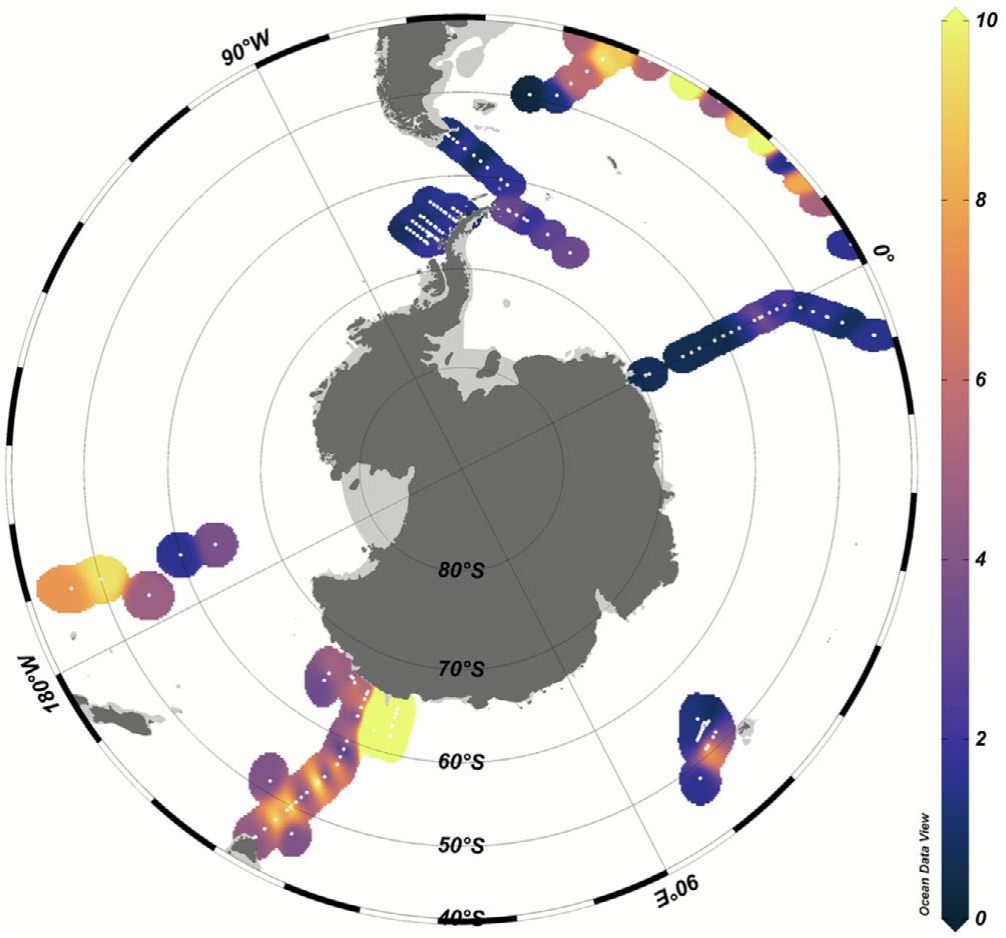
The molar ratio of integrated dissolved Mn to Fe over the upper 100 m. Low values indicate relative depletions in Mn, compared to Fe, while high values indicate a relative surplus in Mn, compared to Fe. Uses data from the 2021v2 GEOTRACES Intermediate Data Product (GEOTRACES‐Intermediate‐Data‐Product‐Group 2021).

The latest CMIP6 ESMs (and those being used for CMIP7 to inform the latest assessments) do not include Mn, and while they all typically include Fe, many struggle to reproduce observed Fe dynamics and neglect newer findings on key processes (Tagliabue et al. 2016; Tagliabue et al. 2023). At present, we lack a complete understanding of how regional trends in Mn deficiency and limitation may operate alongside and interact with those for Fe. Conceptually, the Fe/Mn stoichiometry has proved to be useful in understanding the impacts in experimental and modelling studies (Browning et al. 2021; Hawco et al. 2022; Latour, Strzepek, et al. 2023). Current datasets from the international GEOTRACES programme display significant regional differences in the Fe and Mn inventories of the upper 100 m, with the Atlantic and Pacific sectors being relatively depleted in Mn, as compared to the Indian sector (Figure 7). Interestingly, this matches well with the observed lack of Mn limitation or Mn‐Fe colimitation in the Indian sector (Latour, Strzepek, et al. 2023) and a greater response to Mn in the Drake Passage region (Browning et al. 2021). Nevertheless, there is still a very sparse dataset to work with.

Properly accounting for the roles of Fe and Mn in the context of a changing climate requires an understanding of how each element is coupled and decoupled through both external supply and internal cycling processes. Both Fe and Mn are supplied from the same external sources: natural and anthropogenic aerosols (including fires), continental margin sediments, hydrothermal vents, and the cryosphere, but we lack sufficient constraints on the relative importance and physicochemical speciation of these different sources and their interaction with internal cycling to drive ocean distributions. Ultimately, Fe and Mn distributions will interact with the seasonal mixed layer cycle (Ramalepe et al. 2024; Tagliabue et al. 2014) and supplies at the air‐sea interface (either from aerosols or sea ice/glaciers/ice sheets) to drive the eventual Fe/Mn supply stoichiometry. Aerosol Mn/Fe ratios in the Atlantic sector of the SO are depleted in Mn, but the soluble element ratio is much higher (median 0.31) than the total element ratio (median 0.03) (Chance et al. 2015; Sholkovitz et al. 2012). Future impacts of changing atmospheric supply on Southern Ocean Fe/Mn stoichiometry are therefore potentially susceptible to both total aerosol composition and loading changes, and to relative changes in the fractional solubilities of Fe and Mn. Current models of atmospheric Fe struggle to reproduce the observed fractional solubility of Fe over the Southern Ocean (Ito et al. 2019; Myriokefalitakis et al. 2018) and equivalent models for Mn are only just emerging (Lu et al. 2024).

Arguably, the most challenging aspect of modeling the internal cycling of Fe and Mn in the Southern Ocean is the non‐conservative nature of these metals and their dual roles as both nutrients and scavenged elements. Both are highly particle reactive, with redox chemistry and low solubility leading to short residence times (Tagliabue et al. 2016). This means that dissolved Fe and Mn cannot be modeled in the same way as the longer residence time macronutrients. Advancing our knowledge on future changes requires a better understanding of the similarities and differences in their (i) solubilisation kinetics at source (spanning aerosol deposition, sediment interface, shelf slopes, ice‐ocean interactions, and hydrothermal plumes), (ii) scavenging, colloid production, organic matter associations, photochemistry, and redox kinetics, and (iii) recycling, sinking, and remineralisation through the biogenic, lithogenic, authigenic, and glaciogenic phases that will be important in different parts of the Southern Ocean. Key insights may be gained by coupling water column sampling and particle characterisation with in situ experimental platforms (e.g., Bressac et al. 2019) that can determine the degree to which Fe and Mn are coupled/decoupled through mesopelagic processes and how this is modulated by different particulate phases. Linking this to the prevailing physical regimes (see Section 4.1) will constrain supplies to surface‐dwelling phytoplankton.

### 4.3 Regional Specificities in Phytoplankton Physiology and Zooplankton Recycling

Antarctic phytoplankton display distinct traits, relative to temperate plankton, that reflect their adaptation to the unique Southern Ocean nutrient environment. These include traits that optimise iron costs in photosynthesis and acquisition (Andrew et al. 2023; Hawco et al. 2022; Ryan‐Keogh et al. 2017; Strauss et al. 2023; Strzepek et al. 2019; Strzepek et al. 2012; Strzepek et al. 2011; Sunda and Marchetti 2024), which can contribute to differing responses to climate change (Anugerahanti and Tagliabue 2024). However, existing model assessments assume wholesale changes to global phytoplankton physiology and do not represent the distinct adaptations observed in different regional groups that will have ecological implications in a changing environment. As such, the in situ photosynthetic strategy of Southern Ocean phytoplankton, the resources required to facilitate this strategy in terms of Fe and Mn, and the achieved rates of primary production need to be better understood and integrated into predictive modelling. In addition, while it is assumed that most cellular costs of Fe and Mn are associated with photosynthesis, the role of the management of oxidative stress through the superoxide dismutase (SOD) enzymes may add additional Mn requirements beyond the 4 atoms per PSII in the oxygen evolving complex. SOD demands are expected to rise under Fe stress (Peers and Price 2004), which opens up a potentially important co‐limitation between Fe and Mn (McCain et al. 2021). Efforts to include the Mn cost associated with MnSOD in models find that it can have a significant impact on the ‘footprint’ of Mn limitation, but uncertainties associated with the overall reactive oxygen production rates, the employment of MnSOD versus other forms of SOD that do not require Mn in Mn‐limited regions, and the enzymatic efficiency contribute substantial uncertainty (Anugerahanti and Tagliabue 2023). The specific adaptations within the photosynthetic apparatus of Southern Ocean phytoplankton (Andrew et al. 2023; Hawco et al. 2022; Strauss et al. 2023; Strzepek et al. 2019; Strzepek et al. 2012; Strzepek et al. 2011; Sunda and Marchetti 2024), alongside any sensitivities of oxidative stress with Mn and Fe requirements and availabilities, might also interact with the light field and hence responses to observed deepening trends in summer time Southern Ocean mixed layers (Sallee et al. 2021). Laboratory culture work has also shown how Mn acquisition by temperate phytoplankton can be inhibited by the high levels of free Zn typical of the Southern Ocean (Sunda and Huntsman 1996), if Southern Ocean phytoplankton communities are similarly sensitive, then this trait can play a significant role in shaping regional productivity and carbon export (Hawco et al. 2022). These aspects of the regional ecophysiology‐biogeochemistry have been neglected by ESMs to date, and we require an assessment of their role in governing the across‐model uncertainties in both NPP projections and current trends.

There is growing awareness that zooplankton‐mediated Fe‐cycling plays a crucial role in maintaining productivity in Fe‐limited systems like the Southern Ocean (Boyd et al. 2012; Strzepek et al. 2005). Nevertheless, the representation of this process in ESMs and other global ocean biogeochemistry models remains poorly constrained—if represented at all. The most basic zooplankton model representations assume a fixed rate of Fe‐recycling, an approach that is at odds with the growing empirical data that demonstrate highly variable Fe excretion rates by zooplankton (Chen et al. 2014). Other models allow Fe‐recycling rates to vary through the use of an assumed zooplankton Fe‐quota (see table 1 in Tagliabue et al. 2016). This approach enables models to dynamically respond to changing food conditions and can therefore be used to explore the interactive effects of future climate scenarios. However, the current representation of dynamic Fe‐recycling by zooplankton assumes that Fe‐uptake by zooplankton is optimal and its recycling is lowest when zooplankton biomass stoichiometry equals food stoichiometry (Richon et al. 2020). In reality, this construct will only hold if zooplankton use all elements for growth with equal efficiency; something that we know to be unrealistic because zooplankton use carbon for both energy generation and growth, whereas other elements can be used solely for growth (Hessen and Anderson 2008). The theoretical framework of metabolic stoichiometry (Anderson et al. 2021), in which the fate of ingested macromolecules and their constituent elements are parameterised based on measurable physiological rates, offers great promise for improved realism of zooplankton‐mediated cycling of Fe and Mn. However, adopting this approach requires an improved understanding of zooplankton trace metal stoichiometry and the efficiencies and rates with which they absorb, assimilate, and turnover these elements, which we currently lack.

## 5 Conclusions

An analysis of remote sensing and BGC‐Argo trends in NPP across multiple algorithms reveals a majority view of declines in regional NPP, except for some of the waters near Antarctica. CMIP6 ESMs reveal negligible trends over the recent past and coherent projections of increasing NPP over the coming century. These future trends are surprisingly very insensitive to a range of climate scenarios. Despite similar future outlooks, CMIP6 ESMs show a strong degree of heterogeneity in the role played by primary producer biomass and limitation by both light and iron, indicating poor mechanistic agreement. In parallel, we document an array of key processes around the cycling of multiple limiting nutrients and the regional ecology that are omitted by all ESMs. This indicates we do not have a complete picture of the overall uncertainty from currently available ESM ensembles.

Looking forward, for new knowledge associated with the poorly constrained components of chemical and biological processes to have an impact on model projections, we need a sufficiently complex underlying biogeochemical model architecture. In particular, it is important that any representation of Fe and Mn cycling and biological responses through phytoplankton and zooplankton is parameterised based on assumptions and fluxes that can be constrained through observations and experimentation. Present generation ESMs either do not represent certain specific components (e.g., cycling and biological role for Mn, impact of micronutrients on heterotrophic processes) or do not confer sufficient flexibility/complexity in key areas (e.g., phytoplankton photophysiology and metal demands, zooplankton cycling, role of different Fe and Mn phases through particles and aerosols). These gaps represent important priorities for future research in the region and provide context for policy discussions around advancement of marine protection of Antarctic ecosystems that require sufficiently confident projections of climate change impacts.

## Author Contributions

**Alessandro Tagliabue:** conceptualization, data curation, formal analysis, funding acquisition, investigation, methodology, project administration, resources, writing – original draft. **Thomas Ryan‐Keogh:** conceptualization, data curation, formal analysis, investigation, methodology, software, visualization, writing – review and editing. **Alex Baker:** funding acquisition, writing – review and editing. **Thomas S. Bibby:** funding acquisition, writing – review and editing. **Chris Follett:** funding acquisition, writing – review and editing. **Maeve C. Lohan:** funding acquisition, writing – review and editing. **Alberto Naveira‐Garabato:** funding acquisition, writing – review and editing. **Daniel J. Mayor:** funding acquisition, writing – review and editing. **Angela Milne:** funding acquisition, writing – review and editing. **C. Mark Moore:** funding acquisition, writing – review and editing. **Simon Ussher:** funding acquisition, writing – review and editing.

## Funding

This work was supported by the Natural Environment Research Council, NE/Z000327/1.

## Conflicts of Interest

The authors declare no conflicts of interest.

## Supporting information


**Data S1:** gcb70653‐sup‐0001‐supinfo.docx.

## Data Availability

All data and code will be available at https://zenodo.org/records/17753521.
