# Supplementary material for: The Evolution of Southern Ocean Net Primary Production in a Changing Climate: Challenges and Opportunities
Source: Glob Chang Biol. 2025 Dec 15;31(12):e70653. doi: 10.1111/gcb.70653 (PMC12703219; doi:10.1111/gcb.70653)

s**Supplementary Table 1.** Variables and their data sources used in the calculation of the 6 different remote sensing NPP algorithms. For more details please see Ryan-Keogh et al. (2025).

|  | **Eppley-VGPM** | **Behrenfeld-VGPM** | **Behrenfeld-CbPM** | **Westberry-CbPM** | **Lee-AbPM** | **Silsbe-CAFE** | *Source* |
| --- | --- | --- | --- | --- | --- | --- | --- |
| Chl-a | ✓ | ✓ | ✓ | ✓ |  | ✓ | *OC-CCI* |
| b_bp_ (λ443) |  |  | ✓ | ✓ |  | ✓ | *OC-CCI* |
| a_ph_ (λ443) |  |  |  |  | ✓ | ✓ | *OC-CCI* |
| a_dg_ (λ443) |  |  |  |  |  | ✓ | *OC-CCI* |
| K_d_(λ490) |  |  | ✓ | ✓ | ✓ | ✓ | *OC-CCI* |
| PAR | ✓ | ✓ | ✓ | ✓ | ✓ | ✓ | *GlobColour* |
| SST | ✓ | ✓ |  |  |  | ✓ | *GHRSST* |
| MLD |  |  | ✓ | ✓ |  | ✓ | *Hadley* |
| ZNO_3_ |  |  |  | ✓ |  |  | *WOA2023* |
| SSS |  |  |  |  |  | ✓ | *WOA2023* |
| η |  |  |  |  |  | ✓ | *OC-CCI* |

**Supplementary Table 2.** Variables and their variants downloaded for each CMIP6 Earth system model from the Earth System Grid Federation.

| **Model** | **Variant** | **NPP** | **C_phyto_** | **Fe & Light limitation** |
| --- | --- | --- | --- | --- |
| ACCESS-ESM1-5 | r1i1p1f1 | ✓ | ✓ |  |
| CESM2 | r4i1p1f1 | ✓ |  |  |
| CESM2-WACCM | r1i1p1f1 | ✓ | ✓ | ✓ |
| CMCC-ESM2 | r1i1p1f1 | ✓ |  |  |
| CNRM-ESM2-1 | r1i1p1f2 | ✓ | ✓ | ✓ |
| CanESM5 | r1i1p2f1 | ✓ | ✓ |  |
| GFDL-ESM4 | r1i1p1f1 | ✓ | ✓ | ✓ |
| IPSL-CM6A-LR | r1i1p1f1 | ✓ | ✓ | ✓ |
| MIROC-ES2L | r1i1p1f2 | ✓ | ✓ |  |
| MPI-ESM1-2-HR | r1i1p1f1 | ✓ | ✓ |  |
| MPI-ESM1-2-LR | r1i1p1f1 | ✓ | ✓ |  |
| NorESM2-LM | r1i1p1f1 | ✓ | ✓ |  |
| NorESM2-MM | r1i1p1f1 | ✓ | ✓ |  |
| UKESM1-0-LL | r1i1p1f2 | ✓ | ✓ | ✓ |

**Supplementary Table 3.** NPP means and trends for each remote sensing algorithm. Trends are calculated for both the full time series (1998-2024) and the Jackknife assessments of 80% of the time series. The bgc-Argo subsampled values were calculated by subsampling every year with the unique locations of each bgc-Argo profile, before calculating the climatological mean and trends. Jackknife trend values represent the mean ± the standard deviation of the assessments.

| **Algorithm** | **Climatological Mean**  **(Pg C)** | **Full Time Series Trend**  **(g C m^-2^ year^-1^)** | **Jackknife Mean Trend**  **(g C m^-2^ year^-1^)** |
| --- | --- | --- | --- |
| Eppley-VGPM | 6.89±0.43 | 0.49 | 0.41±0.14 |
| Behrenfeld-VGPM | 9.80±0.58 | 0.70 | 0.63±0.13 |
| Behrenfeld-CbPM | 12.07±0.83 | -0.68 | -0.50±0.71 |
| Westberry-CbPM | 10.78±0.66 | -0.40 | -0.28±0.61 |
| Lee-AbPM | 12.03±0.99 | -0.92 | -1.19±0.28 |
| Silsbe-CAFE | 9.07±0.60 | -0.53 | -0.75±0.26 |
| **bgc Argo subsampled** | | | |
| Eppley-VGPM | 8.01±0.40 | 0.47 | 0.50±0.13 |
| Behrenfeld-VGPM | 12.19±0.57 | 0.70 | 0.77±0.18 |
| Behrenfeld-CbPM | 13.64±1.03 | -0.60 | -0.40±0.77 |
| Westberry-CbPM | 13.13±0.86 | -0.40 | -0.25±0.67 |
| Lee-AbPM | 14.98±1.23 | -1.12 | -1.27±0.32 |
| Silsbe-CAFE | 11.28±0.75 | -0.60 | -0.76±0.31 |

**Supplementary Figure 1** Line plots of input variables to NPP algorithms that have both remote sensing and bcc-argo equivalents, including (a) chlorophyll-a concentrations (Chl-a, mg m-3), (b) phytoplankton carbon (Cphyto, mg m-3), (c) Eppley-VGPM temperature derived photosynthetic rate (PbOpt, μg C (μg Chl-a)-1 h-1), (d) Behrenfeld-VGPM temperature derived photosynthetic rate (PbOpt, μg C (μg Chl-a)-1 h-1), (e) Behrenfeld-CbPM Chl-a:Cphyto derived growth rate (μ, d-1), (f) Westberry-CbPM Chl-a:Cphyto derived growth rate (μ, d-1), (g) VGPM light parameterisation (fPAR, unites), (h) Behrenfeld-CbPM light parameterisation (Φ(I), unitless), (i) Westberry-CbPM light parameterisation (Φ(I), unitless) and the CbPM nutrient-temperature parameterisation (f(N,T), unites). The input variables were spatially averaged across latitude and longitude and resample to annual means. For bgc-Argo[Depth-Weighted] they were integrated over the top 200 m and normalised to this depth range and for bgc-Argo[z < OD1] the mean value in the first optical layer was taken. All remote sensing values are surface only values, except for Westberry-CbPM which is depth-resolved, and the same integration approach was applied as for bgc-Argo. For both VGPM algorithms the fPAR derivation is the same, and for both CbPM algorithms the f(N,T) derivation is the same.

**
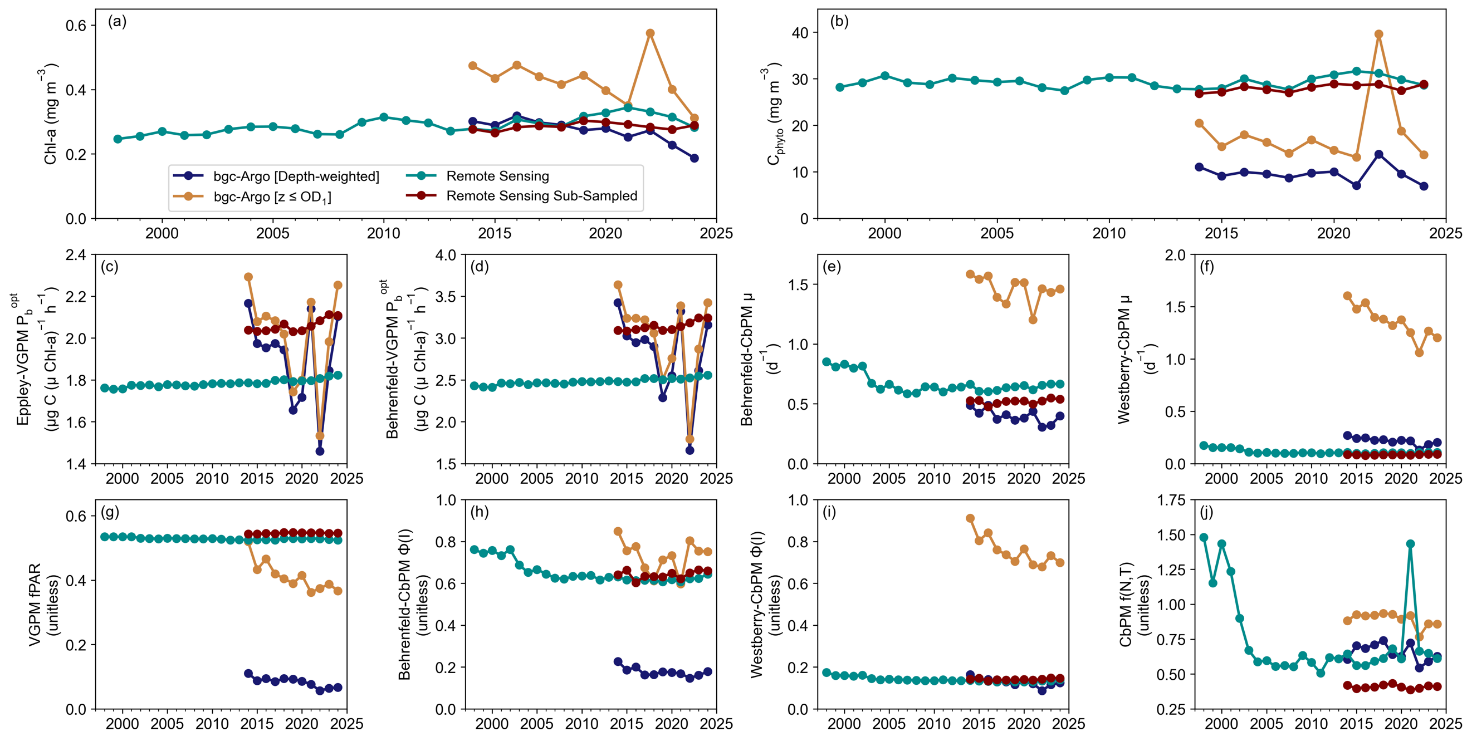
**

**Supplementary Figure 2** A heat map of mean-normalised annual trends (% year-1) for the input variables to NPP algorithms that have both remote sensing and bcc-Argo equivalents. Please see figure S01 for additional details.

**
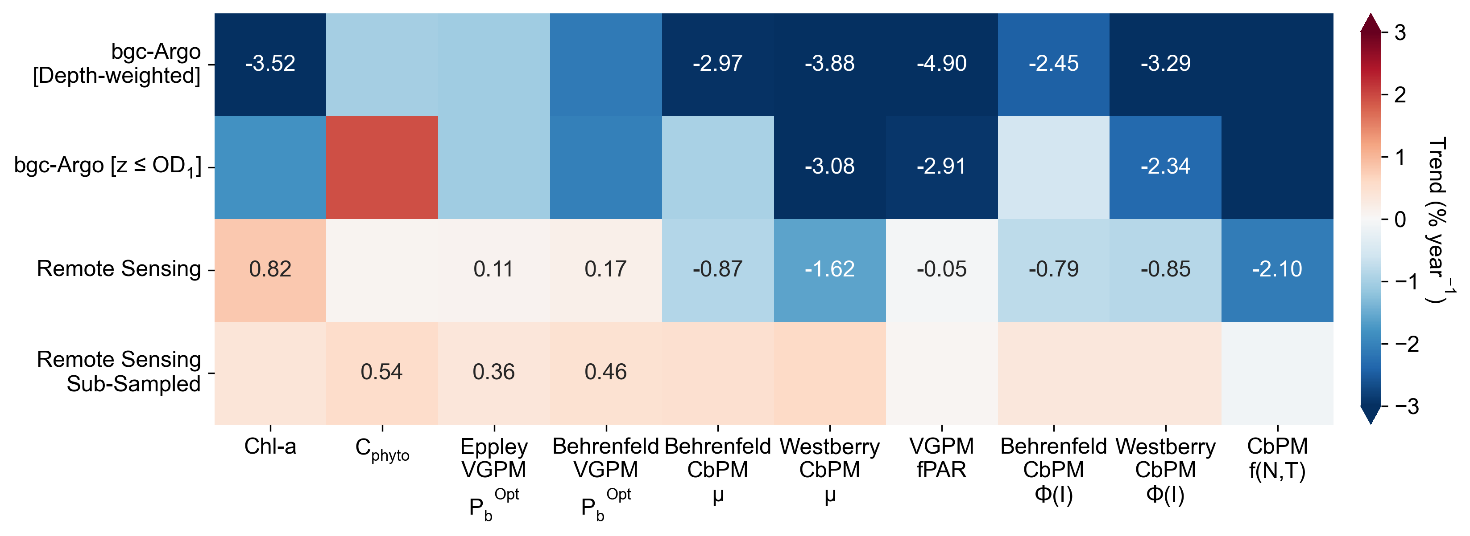
**

**Supplementary Figure 3** Synthesised assessment of how decadal scale changes in NPP relate to those in phytoplankton biomass (C_phyto_), Fe limitation and light limitation for five CMIP6 ESMs between 2005-2014 and 2081-2100 for the SSP2-45 scenario. Symbols represent the mean changes for each model, bars represent one standard deviation across the Southern Ocean region south of 40°S and the colour indicates the strength of the correlation coefficient from a linear regression in space and time of changes in each driver to the change in NPP for each model. Declining limitation terms indicate greater limitation of growth rates.


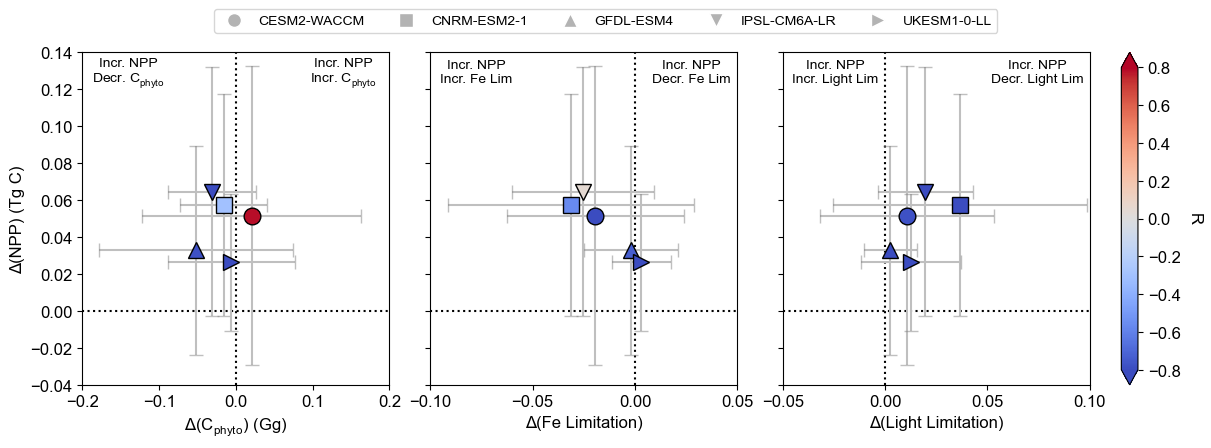


**Supplementary Figure 4** Synthesised assessment of how decadal scale changes in NPP relate to those in phytoplankton biomass (C_phyto_), Fe limitation and light limitation for five CMIP6 ESMs between 2005-2014 and 2081-2100 for the SSP5-85 scenario. Symbols represent the mean changes for each model, bars represent one standard deviation across the Southern Ocean region south of 40°S and the colour indicates the strength of the correlation coefficient from a linear regression in space and time of changes in each driver to the change in NPP for each model. Declining limitation terms indicate greater limitation of growth rates.


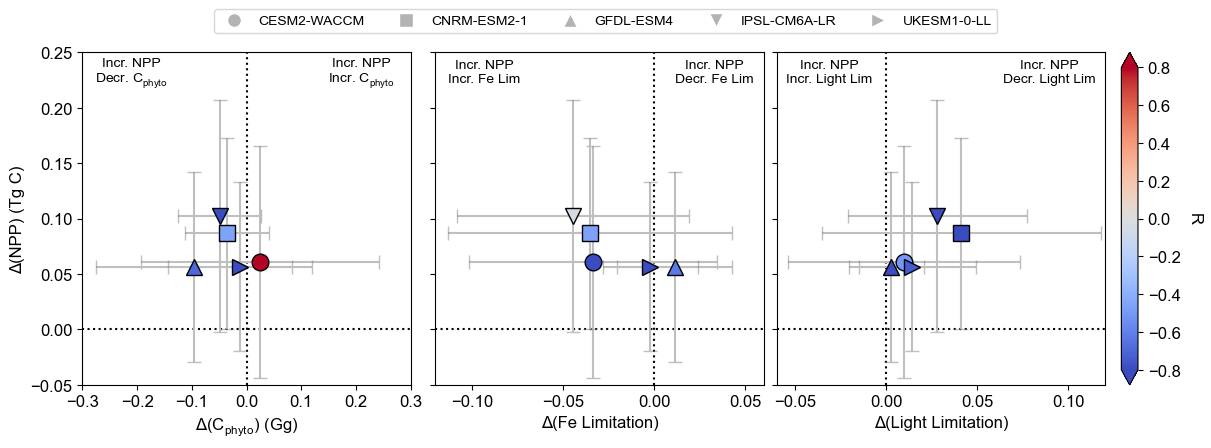


**Supplementary Figure 5** Decadal scale changes in NPP relate to those in phytoplankton biomass (C_phyto_), Fe limitation and light limitation for five CMIP6 ESMs between 2005-2014 and 2081-2100. Declining limitation terms indicate greater limitation of growth rates.


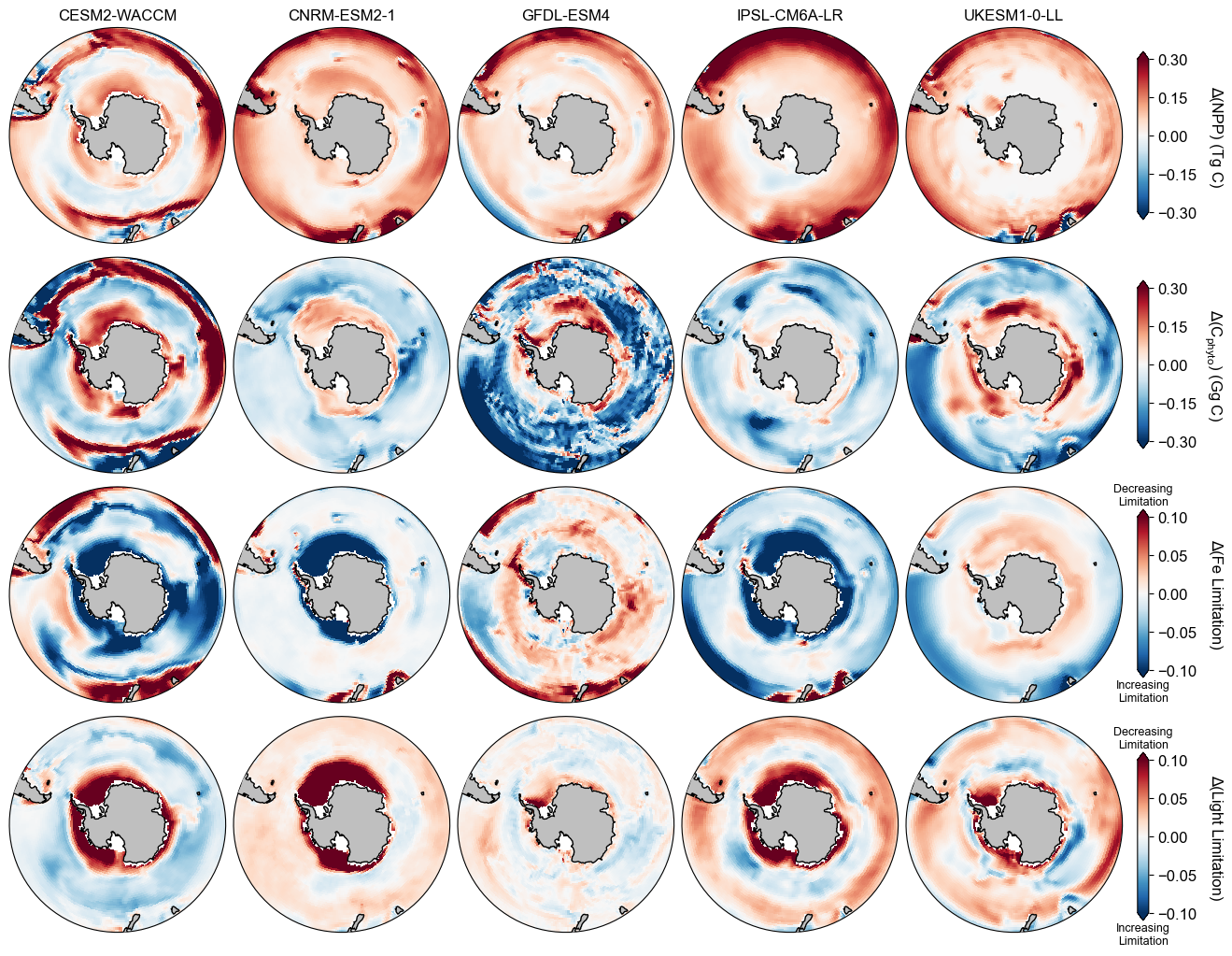

Supplement: Supplementary file 1 — Data S1: gcb70653‐sup‐0001‐supinfo.docx. [file GCB-31-e70653-s001.docx]
